# The Impact of Shared Decision-Making on the Quality of Decision Making in Aortic Dissection: A before-and-after Comparison Study

**DOI:** 10.31083/j.rcm2408244

**Published:** 2023-08-24

**Authors:** Duo Zhang, Haoyang Zheng, Zhi Zheng, Youmin Pan, Zhengbiao Zha, Juan Liu, Lisi Zhu, Qiansheng Wu, Kaili Hu, Zelin Chen, Xiaoxiao Wang, Kan-Paatib Barnabo Nampoukime, Yanrong Zhou

**Affiliations:** ^1^Department of Nursing, Tongji Hospital, Tongji Medical College, Huazhong University of Science and Technology, 430030 Wuhan, Hubei, China; ^2^School of Nursing, Tongji Medical College, Huazhong University of Science and Technology, 430030 Wuhan, Hubei, China; ^3^Department of Neurosurgery, Union Hospital, Tongji Medical College, Huazhong University of Science and Technology, 430022 Wuhan, Hubei, China; ^4^Division of Cardiothoracic and Vascular Surgery, Sino-Swiss Heart-Lung Transplantation Institute, Tongji Hospital, Tongji Medical College, Huazhong University of Science and Technology, 430030 Wuhan, Hubei, China; ^5^Department of Virology, Research Institute for Microbial Diseases, Osaka University, 565-0871 Suita, Osaka, Japan

**Keywords:** shared decision-making, patient decision aids, aortic dissection, surgery, before-and-after comparison study

## Abstract

**Background:**

Complex surgical plans and consideration of risks and benefits often cause decisional conflicts for decision-makers in aortic dissection (AD) surgery, resulting in decision delay. Shared decision-making (SDM) improves decision readiness and reduces decisional conflicts. The purpose of this study was to investigate the impact of SDM on decision quality in AD.

**Methods:**

One hundred and sixty AD decision-makers were divided into two groups: control (n = 80) and intervention (n = 80). The surgical plan for the intervention group was determined using patient decision aids. The primary outcome was decisional conflict. Secondary outcomes included decision preparation, decision satisfaction, surgical method, postoperative complications, actual participation role, and duration of consultation. The data were analyzed with SPSS 26.0 (IBM Corp., Chicago, IL, USA). *p* < 0.05 was considered statistically significant.

**Results:**

The decisional conflict score was significantly lower in the intervention group than in the control group (*p* < 0.001). The decision preparation and decision satisfaction scores in the intervention group were significantly higher than those in the control group (*p* < 0.001). There were more SDM decision-makers in the intervention group (16 [20%] vs. 42 [52.50%]). There was no statistical significance in the choice of surgical, postoperative complications, duration of consultation, and hospital and post-operative intensive care unit stay time (*p* = 0.267, *p* = 0.130, *p* = 0.070, *p* = 0.397, *p* = 0.421, respectively). Income, education level, and residence were the influencing factors of decision-making conflict.

**Conclusions:**

SDM can reduce decisional conflict, improve decision preparation and satisfaction, and help decision-makers actively participate in the medical management of patients with AD without affecting the medical outcome.

## 1. Introduction

Aortic dissection (AD) is a serious life-threatening cardiovascular disease, which has garnered much attention in recent years. AD has an acute onset and a variety of initial symptoms. The incidence is approximately 6/100,000, and the mortality rate is second only to acute myocardial infarction [1]. With the development of medicine and biotechnology, the treatment of AD is a long-term dynamic clinical exploration and practice process that includes thoracotomy, minimally invasive surgery, hybrid surgery, and other treatment schemes. It cannot be ignored that the treatment decisions for either type A or type B AD are risky decisions made in a limited time, because regardless of which treatment is chosen, patients may have risks of bleeding, pain, AD rupture, and reoperation, among others [1]. In addition, most AD patients are in a sedative and analgesic state before surgery, making them lose decision-making ability; thus, their medical decisions are mostly made by family members [2]. Affected by the uncertainty of disease trajectory and individual differences, most AD decision-makers have negative emotions such as anxiety and helplessness [3]. Our previous study showed that approximately 99.09% of AD patients and 98.91% of their family members had decisional conflicts, which were not related to the type of AD [2, 4].

The decision-making of AD is complex, and not only requires doctors to inform disease information within a limited time but also needs consideration of patients’ views and other nonmedical factors. The guidelines for the diagnosis and management of aortic disease jointly issued by the American College of Cardiology/American Heart Association strongly recommend that patients and medical staff jointly decide on treatment plans to determine the endoluminal surgery, thoracotomy, hybrid surgery, etc. [5] Shared decision-making (SDM) is key to improving the quality of decision-making and is a concrete embodiment of “patient-centered” care in clinical practice. SDM is a process by which medical staff and patients work together to integrate care plans that are responsive to patients’ goals and values [6]. It has been advocated as a clinical counseling approach that improves disease knowledge, and reduces anxiety and decisional conflict by encouraging patients to participate in clinical decision-making [7].

At present, SDM has been widely applied to the decision-making process of patients and their surrogate decision-makers in orthopedics [8], cancer [9], and so on, but there are few reports on critical cardiovascular diseases [10, 11]. Under the guidance of the Ottawa Decision Support Framework (ODSF) and the International Patient Decision Aid Standards (IPDAS), we developed a patient decision aid (PtDA) for AD decision-makers. We used PtDA on admission day, preoperative conversation and discharge day, which we termed a “patient-centered SDM”, to be used as part of the medical decision-making of AD. The whole decision-making process was jointly performed by doctors, nurses, patients and their family members with a clear division of labor. The primary objective of this study was to assess the impact of SDM on the decisional conflict of AD decision-makers. Secondarily, this study quantified differences between intervention and control groups on the decision preparation, satisfaction, participation role, final surgical method, postoperative complications, duration of consultation, post-operative intensive care unit (ICU) stay time and hospital stay time.

## 2. Methods

### 2.1 Study Design and Setting

A single-center, before-and-after comparison study of SDM for AD decision-makers was conducted from March 2021 to June 2022, after approval from the Research Ethics Committee of Tongji Medical College, Huazhong University of Science and Technology (s146; Wuhan, Hubei Province, China). We conducted the study in the Department of Cardiovascular Surgery, Tongji Hospital (Wuhan, Hubei Province, China). The annual operation volume of AD was 1000–1200, and most patients were from different parts of China. Our research team included five cardiac surgeons, four SDM experts, three cardiac surgery nurses, and two information and knowledge translation specialists. The whole process of this study was completed by team members without blinding.

### 2.2 Participants

The uncertainty of the development of AD makes it difficult to recruit participants by phone or email. We allocated AD decision-makers from March to June 2021 to the control group and from March to June 2022 to the intervention group through convenience sampling. Our study object was AD decision-makers, including not only patients but also surrogate decision-makers. Inclusion criteria for patients were: diagnosed with aortic AD, including type A AD and type B AD; age ≥18 years; had a clear consciousness, good communication, and writing skills; and participated in preoperative conversations and signed informed consent forms. It was difficult to achieve effective communication between doctors and patients who needed emergency surgery and were not accompanied by their families. Therefore, they were excluded from our study.

Medical decisions for limited/incapacitated aortic coarctation patients were often made by surrogate decision-makers, who were the legal guardian of patients. Inclusion criteria for the surrogate decision-makers were: age ≥18 years; knew the patient’s diagnosis; good communication skills and writing ability; and participated in the preoperative conversation and signed informed consent forms. We excluded decision-makers who showed preoperative refusal of treatment and had unresolved conflicts with medical staff. In addition, we excluded special cases such as AD during pregnancy. On the one hand, this situation was relatively rare. The decision content was not treatment of a single AD but may involve the priority of various diseases and medical decisions under complex situations [12]. On the other hand, similar situations required the cooperation of different medical departments, which was beyond the scope of our PtDA.

### 2.3 Interventions

#### 2.3.1 Intervention Group

Participants in the intervention group received SDM that involved the use of a PtDA booklet developed by the researchers by referring to the ODSF and IPDAS [13, 14]. This tool is an available booklet that is designed for AD decision-makers to choose a treatment plan (**Supplementary Material**).

**Fig. 1.**
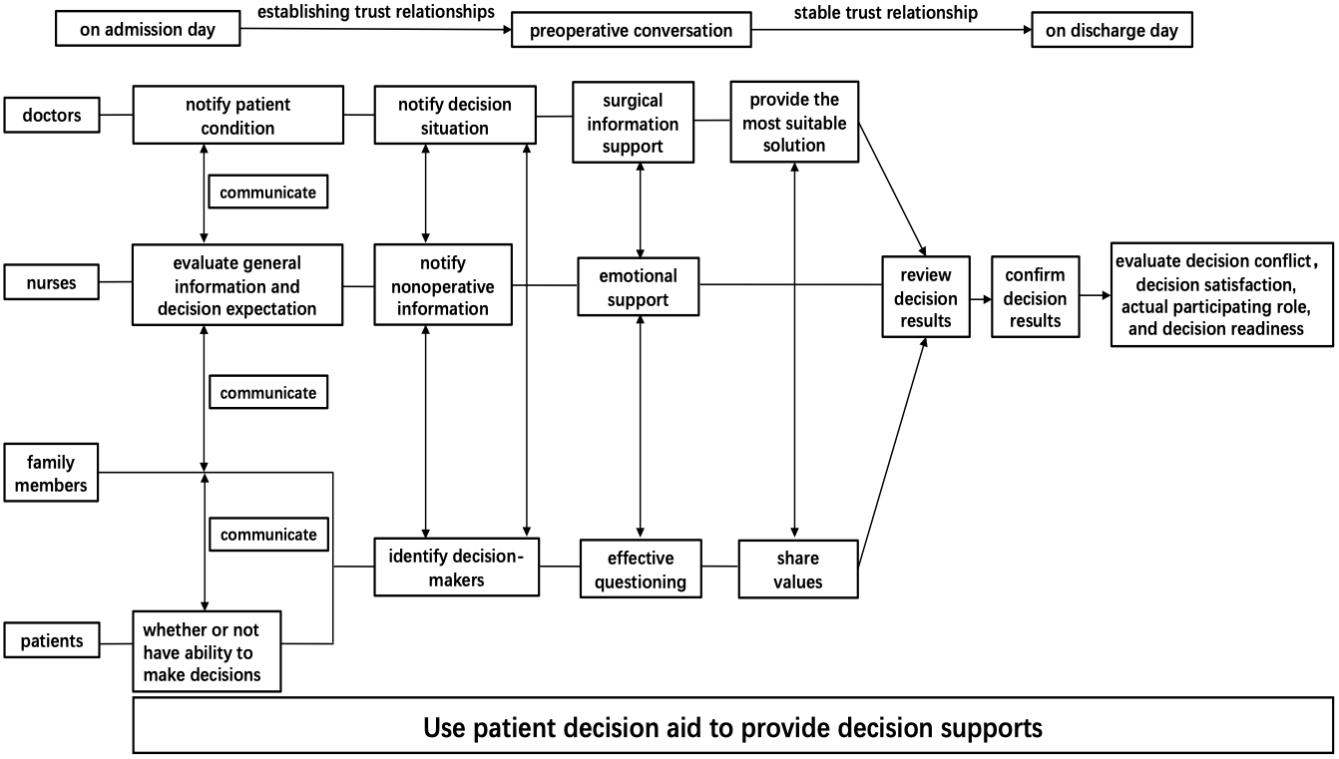
**Flowchart of shared decision-making in the intervention group**.

**Fig. 2.**
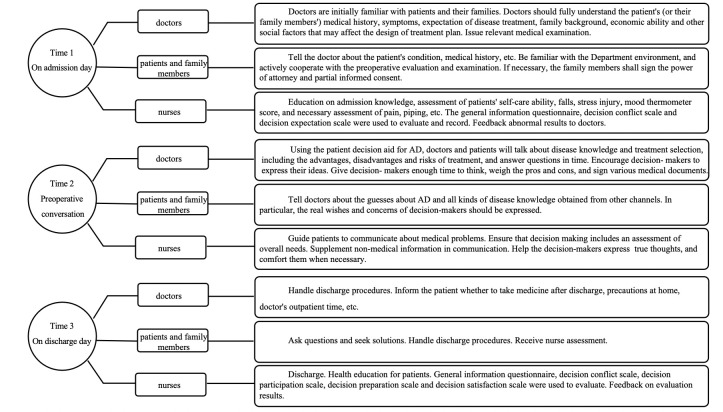
**The tasks of doctors, patients and family members, and nurses at different time points**. AD, aortic dissection.

The intervention group emphasized “patient-centered” SDM, including four contents (Fig. 1). First, identifying current decision needs by recording the disease diagnosis, and judging whether the patient has decision-making ability and decision-making type, etc. Second, providing necessary information including the definition, epidemiological characteristics, clinical manifestations, and treatment principles of AD. We used three simple questions to evaluate the decision-makers’ psychological status, social support, and views on the treatment plans. Third, evaluating the decision-makers’ expectations of treatment results and their acceptance of risks. Finally, the decision-makers evaluated the decision-making process and selected the treatment plan. The whole decision-making process was jointly performed by doctors and nurses with a clear division of labor (Fig. 2). There was no follow-up.

#### 2.3.2 Control Group

Participants in the comparison group received patient education with standard educational material on AD, which contained textual and pictorial information on the definition, diagnosis, epidemiological characteristics, and postoperative health guidance of AD. The doctor explained the operational risks and benefits to the decision-maker, and finally decided the surgery plans. Nurses had minimal interaction with the participants and did not perform teach-back or monitor comprehension in the process. Therefore, the participants did not raise questions or verbalize their concerns; consequently, their values, feelings, and thoughts on the material were not explored.

### 2.4 Procedure

**Fig. 3.**
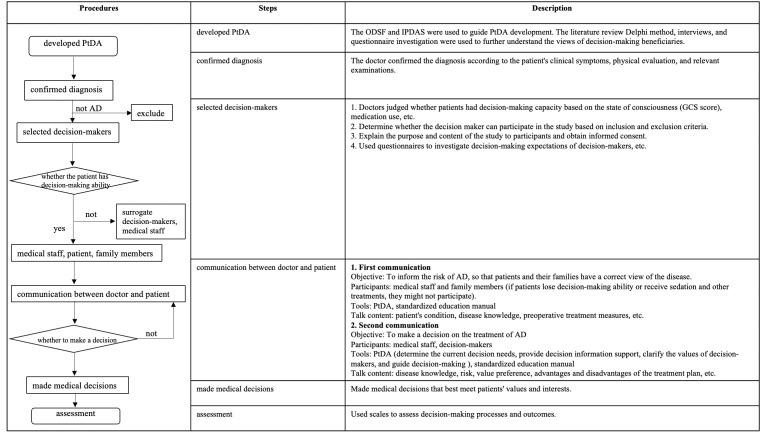
**The content of the procedures**. PtDA, patient decision aid; AD, aortic dissection; ODSF, Ottawa Decision Support Framework; IPDAS, International Patient Decision Aid Standards; GCS, Glasgow Coma Scale.

The two groups of subjects were investigated from March to June 2021 and from March to June 2022, and did not interfere with each other. Clinical staff screened the patients after admission and explained the purpose, procedures, risks, and benefits of the study, after which written informed consent was obtained from the participants. We had our first conversation on the day of admission. Doctors used PtDA to introduce the patient’s condition to the decision-makers of the intervention group, mainly including the definition, risk, and pre-operative treatment measures, etc. of AD. The decision-makers in the control group received the contents from the standardized health education sheet. Although most AD patients needed to receive surgical treatment, not all patients could receive it immediately due to factors such as physical evaluation and other surgical arrangements in the operating room [15]. Even in direct circumstances, there is usually time to have some discussion with patients and surrogates that adheres to the goals of SDM [10]. The second conversation was usually the day before the operation. All participants were interviewed by doctors. The preoperative conversation was completed in the conference room. The control group received the routine procedure. The intervention group received the SDM on the basis of understanding the content of PtDA. The decision-makers continued to communicate with the medical staff until the questions were resolved. We used a stopwatch to record the time from the start of preoperative conversation to signing of the informed consent form. Decision-makers completed the scales such as decision satisfaction on the day of discharge. Fig. 3 shows the content of the procedures.

### 2.5 Measure

#### 2.5.1 Sociodemographic Characteristics

This section was designed by the researchers and included sex, age, decision-makers, habitation, education, marital status, and income.

#### 2.5.2 Primary Outcome Measure

Decisional conflict: The Decisional Conflict Scale (DCS) prepared by O’Connor in 1995 (Cronbach’s α = 0.78–0.92) [16], is often used to identify patients’ decision support needs, judge the quality of the decision-making process, and evaluate the effects of decision support intervention. There are 16 items in the DCS including three aspects: decision uncertainty, decision uncertainty factors, and perceived decision effectiveness. The scale is a 5-point Likert scale (ranging from 0 to 4). The higher the total score, the more serious the decisional conflict. In this study, we evaluated decisional conflict using the modified Chinese version of the DCS developed by Wang *et al*. [17] on discharge day. The Chinese version showed high internal consistency (Cronbach’s α = 0.886).

#### 2.5.3 Secondary Outcome Measures

Decision preparation: The Preparation for Decision-Making (PreDM) scale was prepared by Bennett *et al*. [18] (Cronbach’s α = 0.92–0.96). There are 10 items in the PreDM, which are mainly used to evaluate the preparation of PtDA to help decision-makers communicate with medical staff in the decision-making process. It is a 5-point Likert scale (ranging from 1 to 5). The higher the total score, the better the decision-making preparation. In this study, we evaluated decision preparation using the modified Chinese version of PreDM developed by Li [19] on discharge day. The Chinese version showed high internal consistency (Cronbach’s α = 0.946).

Decision satisfaction: This scale was prepared by Xu (Cronbach’s α = 0.899) [20]. There are 16 items in the scale including four aspects: information, communication, decision-making, total satisfaction and confidence. It is often used to measure the degree of satisfaction in surgical decision-making. The scale uses a 5-point Likert scale (ranging from 1 to 5). The higher the total score, the higher the decision-makers’ satisfaction with decision-making.

Participation role: The Control Preferences Scale (CPS) was prepared by Degner *et al*. [21] (Cronbach’s α = 0.5–0.91). The scale consists of five options A–E, in which A or B represents active decision-making (patients make decisions independently), C represents SDM (medical staff and patients collaborate to make a medical decision), and D or E represents passive decision-making (doctors help patients make decisions). The CPS is often used to investigate subjects’ tendencies and actual participation in the process of medical decision-making. In this study, we evaluated the decision participation role using the modified Chinese version of CPS developed by Peng [22] on admission and discharge days. The Chinese version showed high internal consistency (Cronbach’s α = 0.36–0.91).

In addition to the above scales, we included the final surgical method, postoperative complications, duration of consultation in minutes, hospital stay time, and post-operative ICU stay time in the secondary measures to explore the impact of SDM.

### 2.6 Target Sample Size

Our study evaluated the impact of SDM on decision quality. The decisional conflict score was the primary outcome index [23]. There was no relevant study on the decision-making of AD; thus, we calculated the sample size according to the results of the pre-experiment. The survey results of two groups reported that the decisional conflict scores were 30.20 ± 5.574 and 34.80 ± 8.638. According to a previous study [24], we set the test of type α error = 0.05, 1–β error = 0.95, and allocation ratio = 1:1. The sample size of 132 subjects (n = 66 in each group) in the study was estimated using G-Power software 3.1.9.6 (Heinrich Heine University, Dusseldorf, North Rhine-Westphalia, GER). Taking into account the 10% probability of loss, the sample size was increased to 73 in each group. For the convenience of calculation, 90 decision-makers of AD surgery were included in each group.

### 2.7 Statistical Analysis

We used SPSS 26.0 (IBM Corp., Chicago, IL, USA) for statistical analysis. Histograms, P-P diagrams, and Q-Q diagrams were used to comprehensively assess whether the data were a normal distribution. Continuous normally distributed variables are expressed as the mean ± standard deviation (SD), and abnormally distributed variables are expressed as the interquartile range. Categorical variables are expressed as numbers and percentages. Continuous normally distributed variables were tested for differences between groups using independent *t*-tests. Categorical variables were tested by the chi-square test and Fisher’s exact test. The Mann-Whitney U test was used for abnormally distributed variables. To understand the relevant factors of decisional conflict, we used multiple stepwise regression analyses to deal with the variables. All statistical tests were two-sided, and *p* < 0.05 was considered statistically significant.

## 3. Results

**Fig. 4.**
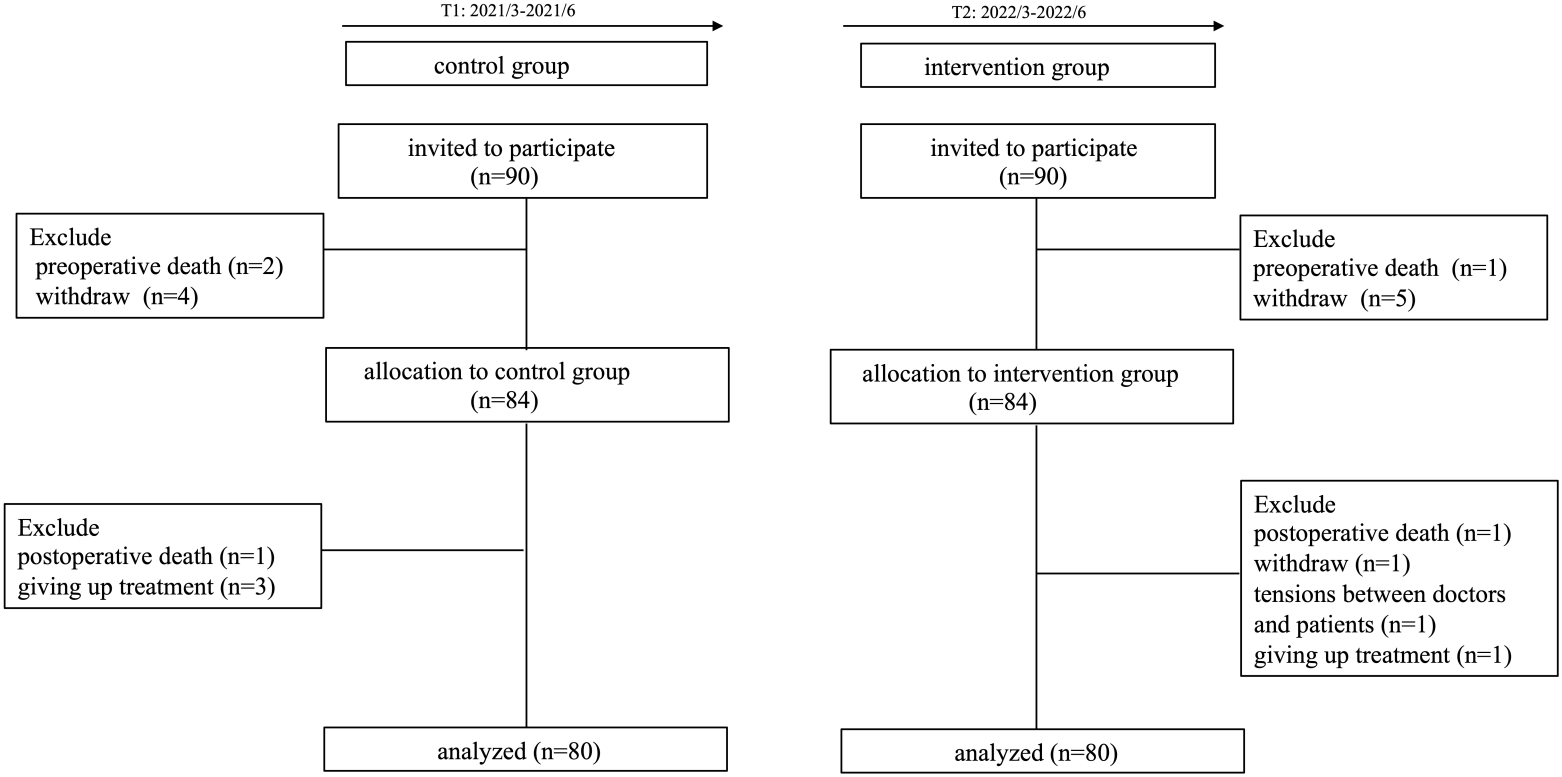
**Patient flowchart**.

Ten participants were excluded from the intervention group due to different reasons, including preoperative death (n = 2), tensions between doctors and patients (n = 1), giving up treatment (n = 1), and withdrawal (n = 6). Moreover, 10 cases were ruled out from the control group due to different reasons, including preoperative death (n = 3), giving up treatment (n = 3), and withdrawal (n = 4). Finally, 160 people were included in the study (Fig. 4).

### 3.1 Baseline Characteristics

In total, 80 decision-makers of AD were included in the intervention group and the control group. There were no significant differences in sociodemographic characteristics between the two groups (Table 1).

**Table 1.** **Baseline characteristics of AD decision-makers (n = 160)**.

Variable		Control group (n = 80)	Intervention group (n = 80)	*p* value
Sex				0.465*
	Male	22 (27.50%)	18 (22.50%)	
	Female	58 (72.50%)	62 (77.50%)	
Age				0.356*
	<40	17 (21.30%)	15 (18.80%)	
	40–60	52 (65.00%)	47 (58.80%)	
	>60	11 (13.80%)	18 (22.50%)	
Decision-makers				0.059*
	Patients	30 (37.50%)	19 (23.80%)	
	Proxy decision-makers	50 (62.50%)	61 (76.30%)	
Residence				0.103*
	Rural	64 (80.00%)	55 (68.80%)	
	Urban	16 (20.00%)	25 (31.30%)	
Education				0.591*
	Primary school and below	13 (16.30%)	9 (11.30%)	
	Junior middle school	31 (38.80%)	38 (47.50%)	
	High school/junior college	25 (31.30%)	25 (31.30%)	
	Bachelor’s degree or above	11 (13.80%)	8 (10.00%)	
Marital status				0.416*
	Married	71 (88.75%)	74 (92.50%)	
	Others	9 (11.25%)	6 (7.50%)	
Income				0.358*
	<3000	12 (15.00%)	9 (11.30%)	
	3000–6000	35 (43.80%)	44 (55.00%)	
	>6000	33 (41.30%)	27 (33.80%)	
Expected participation role				0.712**
	Active decision-making	1 (1.25%)	3 (3.75%)	
	SDM	40 (50.00%)	40 (50.00%)	
	Passive decision-making	39 (48.75%)	37 (46.25%)	

The number or number (percentage) is shown; AD, aortic dissection; SDM, shared decision-making. *chi-square test, ** Fisher’s exact test.

### 3.2 Primary Outcome Measures

Table 2 shows the difference in decisional conflict scores between the intervention group and the control group. Compared with the control group, the decision-making conflict score in the intervention group was lower and a significant difference was observed between groups (*p* < 0.001). The scores of subscales (decision uncertainty, decision uncertainty factors, perceived decision effectiveness) were also lower in the intervention group compared to those obtained in the control group. However, there was no significant difference in perceived effectiveness on the subscale (*p* = 0.058).

**Table 2.** **Comparison of decisional conflict scores between the two groups**.

Variable	Control group (n = 80)	Intervention group (n = 80)	*p* value
Decisional conflict	35.33 (5.74)	32.04 (4.74)	<0.001*
Decision uncertainty	7.41 (1.87)	6.34 (1.28)	<0.001*
Decision uncertainty factors	20.04 (4.05)	18.40 (3.23)	0.005*
Perceived decision effectiveness	8.00 (6.00, 9.00)	7.00 (6.00, 9.00)	0.058**

The mean ± standard deviation or interquartile is shown. * independent *t*-test, ** Mann-Whitney U test.

### 3.3 Secondary Outcome Measures

Table 3 shows the score difference in secondary outcome measures between the intervention group and the control group. Compared with the control group, the decision-making preparation and satisfaction of the intervention group were significantly improved (*p* < 0.001). The implementation of SDM effectively improved the actual participation role of decision-makers (*p* < 0.001), enabled more decision-makers to participate in preoperative conversations (SDM number [percentage], 16 [20%] vs. 42 [52.50%]), and reduced passive decision-making (passive decision-making number [percentage], 62 [77.50%] vs. 36 [45.50%]). However, the SDM did not change the patient’s choice of final treatment plans or the occurrence of postoperative complications (*p* = 0.267 and *p* = 0.130, respectively). No significant difference was found in the duration of encounters between the intervention and control groups (*p* = 0.070). No significant difference was found in the hospital and post-operative ICU stay time between the two groups (*p* = 0.421).

**Table 3.** **Comparison of scores in secondary outcome measures between the two groups**.

Variable		Control group (n = 80)	Intervention group (n = 80)	*p* value
Decision preparation		25.43 (2.04)	32.39 (2.95)	<0.001*
Decision satisfaction		46.81 (5.22)	50.30 (3.59)	<0.001*
Information		11.08 (1.89)	12.56 (2.18)	<0.001*
Communication		12.37 (2.25)	12.24 (1.83)	0.672*
Decision-making		9.20 (1.63)	10.11 (1.88)	0.001*
Total satisfaction and confidence		14.16 (2.59)	15.38 (2.05)	0.001*
Actual participation role				<0.001**
	Active decision-making	2 (2.50%)	2 (2.50%)	
	SDM	16 (20.00%)	42 (52.50%)	
	Passive decision-making	62 (77.50%)	36 (45.50%)	
Whether the decision maker’s expected participation role is consistent with the actual participation				1.000***
	Yes	33 (41.25%)	33 (41.25%)	
	No	47 (58.75%)	47 (58.75%)	
Final treatment plans				0.267***
	Thoracotomy	22 (27.50%)	17 (21.30%)	
	Minimally invasive surgery	49 (61.30%)	47 (58.80%)	
	Hybrid surgery	9 (11.30%)	16 (20.00%)	
Postoperative complications				0.130***
	No	66 (82.50%)	58 (72.50%)	
	Yes	14 (17.50%)	22 (27.50%)	
Duration of encounter, interquartile, min		33.00 (30.00, 37.75)	33.00 (29.25, 35.75)	0.070****
Hospital stay time		16.26 (3.05)	15.79 (3.97)	0.397*
Post-operative ICU stay time		3 (2, 6)	5 (2.25, 6)	0.421****

Data are expressed as the mean ± standard deviation, interquartile or number (percentage); SDM, shared decision-making; ICU, intensive care unit. * independent *t*-test, ** Fisher’s exact test, *** chi-square test, **** Mann-Whitney U test.

### 3.4 Multiple Stepwise Regression Results of Decisional Conflict

Significant variables in univariate analysis of residence (*p* < 0.001), education levels (*p* < 0.001), and income (*p* < 0.001) were included in the multiple stepwise regression analysis. The dummy variable was set for residence. Significant independent factors influencing the DCS score were income, education levels, and residence (Table 4). Higher income and education levels, and living in urban areas led to lower decision-making conflicts among AD decision-makers.

**Table 4.** **Results of multiple stepwise regression analysis related to decisional conflict (n = 160)**.

Variable		β	*SE*	β’	*t*	*p* value
(Constant)		43.749	0.776		56.392	<0.001
${\mathrm{Income}}^{1}$						
	3000–6000	–5.650	0.830	–0.515	–6.811	<0.001
	>6000	–7.969	0.987	–0.704	–8.077	<0.001
${\mathrm{Education}}^{2}$						
	Junior middle school	–3.072	0.811	–0.278	–3.787	<0.001
	High school/junior college	–4.996	0.968	–0.422	–5.159	<0.001
	Bachelor’s degree or above	–7.364	1.253	–0.435	–5.877	<0.001
${\mathrm{Residence}}^{3}$ (urban)		–2.064	0.723	–0.164	–2.856	0.005

$R^{2}$ = 0.697, after adjustment $R^{2}$ = 0.686; F = 8.157, *p* < 0.05. ^1^ Reference: <3000. ^2^ Reference: primary school and below. ^3^ Reference: rural.

## 4. Discussion

Heart and macrovascular diseases are important areas of SDM. We developed a PtDA for AD incorporating decision needs, patient education, preference assessment, and personalized estimations of clinical outcomes, which were presented in the form of words, tables and pictures. We used PtDA in the SDM of AD, defined the tasks of doctors, nurses, patients and family members, and emphasized building trust relationships between medical staff and patients in the process of communication. Compared with traditional preoperative conversations, this study evaluated the impact of SDM on the outcome of AD surgery. Our study demonstrated that the implementation of SDM for patients undergoing AD surgery was possible and effective in our institution. SDM was capable of improving the quality of decision-making without changing the choice of surgical methods or impacting medical outcomes.

Decisional conflict is a state of uncertainty in the course of action that exists and permeates the decision-making process of AD, increasing the pressure on decision-makers [25]. Previous studies have shown that for each unit increase in DCS score, decision-makers are 59 times more likely to change their minds and 23 times more likely to delay their decisions [26, 27]. AD surgery is risky and uncertain, and the delay in treatment leads to increased complications, which greatly increases the risk of death [28]. Encouragingly, we found that SDM could reduce decisional conflict, which was consistent with the results of previous randomized controlled trials [29]. Subscale analysis showed that decision uncertainty and decision uncertainty factors scores compared between the two groups were statistically significant (*p* < 0.001, *p* = 0.005). The AD PtDA is comprehensive, objective, and fair. It provides disease information and stress relief methods that are practical needs, which can improve patients’ and families’ knowledge of disease and surgical risks, and reduces the influence of uncertainty factors on the decision-making process. The perceived effectiveness of the two groups was not statistically significant (*p* = 0.058). This is because AD decisions are made before surgery, and medical staff and decision-makers cannot guarantee that no risks will arise during the procedure. Uncertainty about disease risk leads to a lack of confidence in the results of decision-making [30]. Some studies have indicated that SDM is not significant in reducing decisional conflict [31]. This may be related to disease characteristics, health literacy, etc. [32]. The effects of objective and subjective health literacy on patients’ accurate judgment of health information need to be investigated in the future.

Decision control preference reflects the desire of patients and their families to make decisions independently. The results showed that the actual decision-making participation in the control group was mostly passive decision-making (77.50%), and the intervention group was SDM (52.50%). Low decision control preference means high treatment expectations [33]. SDM reduces the gap between the expectation and reality of surgical results, and attaches importance to the doctor-patient relationship based on trust. In the PtDA, we sorted out and objectified the issues most concerned by decision-makers and encouraged them to actively ask questions, enhancing their perception of decision-making participation. The PtDA was an optimized logical path that included four steps: determining the current decision needs, providing decision information support, clarifying the values of the decision-makers, and guiding decision-making, which can help decision-makers choose options consistent with their values according to a fixed process and help them realize the situation they are facing. In this study, the use of SDM improved the decision readiness of participants. We used PtDA on the admission day to assess the decision needs of patients and help them gain an initial understanding of AD. During the preoperative conversation, we present the pros and cons of various treatment options in the form of drawings and tables to enhance their understanding of AD, and encourage them to express values. A systematic review in 2016 showed that using SDM could improve decision-makers’ confidence and promote a positive healthcare experience and decision-making process, regardless of their final surgical decision [34].

It should be noted that the core of high-quality decision-making is that the results are consistent with patients’ values, goals, and preferences. Increasing knowledge alone is not enough to make high-quality decisions, especially emotional decisions about life and death [35]. Similarly, encouraging the decision-maker to determine the surgical plans in fear and denial cannot guarantee satisfactory results. After the intervention, the total score of decision satisfaction was significantly improved, especially the information and decision subscale, consistent with the results of Alden [36]. On the one hand, the PtDA for AD improved the decision makers ability to grasp disease knowledge and reduced the inner fear caused by lack of information. On the other hand, in the process of intervention, medical staff respected patients and encouraged them to express their values, which helps to build trust and improve decision-making satisfaction.

We also found that the use of PtDA did not improve the communication between doctors and patients. With the rapid development of AD, decision-making time is limited, and it is difficult to ensure timely communication between doctors and patients. Nurses have the longest contact with patients and their families. With the transformation of nurses’ functions and their prominent role in the SDM process, nurses can transmit information and improve the efficiency of communication.

In contrast to some research results, our study did not find that SDM changed patients’ choice of surgical plans and postoperative situation [37]. At the same time, it did not shorten the time of hospitalization and stay in ICU. For AD patients, the survival advantage of surgery is certain. The choice of surgical plans and the occurrence of complications is affected by medical conditions, such as surgical techniques and basic conditions of patients. The time of hospitalization and stay in ICU are also affected by the operation effect [38]. Although PtDA have deepened the understanding of disease knowledge of decision-makers, enabling them to view the occurrence of risks objectively and rationally, they cannot change the medical outcomes of patients. In addition, the use of PtDA did not have a significant impact on the duration of the conversation, which was consistent with the results of Kunneman *et al*. [39]. PtDA optimizes and supplements the content of informed consent, but does not simplify the medical decision-making process. The SDM 3 Circle Model, the three-stage conversation model, and the SDM model mediated by the decision-making coach, were used to improve the decision-making efficiency [40]. In the future, similar theories can be combined to optimize the intervention process, shorten the preoperative talk time, and improve the quality of decision-making.

Income, education, and residence were the main influencing factors of decisional conflict. In China, the median hospitalization cost for patients with acute AD was as high as 115,296 RMB [41]. Restrictions on medical insurance, post-discharge medication, and rehabilitation, etc., place greater financial pressure on AD patients. Although SDM has been widely used in the clinic, high-income decision-makers have a relatively light economic burden, fewer adverse emotions, and more firm decision results. Highly educated decision-makers have high health literacy and the ability to acquire and understand disease knowledge [42]. They can more effectively receive the information transmitted by medical staff and make medical decisions. Compared with urban patients, the lack of knowledge and medical resources may cause decision-making conflict among rural patients.

## 5. Limitations

To the best of our knowledge, this was the first study to assist decision-makers in participating in the SDM of AD patients through the PtDA. The results was gratifying, which proved the feasibility and effectiveness of PtDA in AD patients. However, some limitations need to be considered. First, the study was conducted in a relatively developed city in China, with strict inclusion and exclusion criteria, which may limit the generalizability of the research results. Second, we could not measure the subjects’ mastery of disease knowledge due to the lack of an AD knowledge scale. Third, we only evaluated each decision-maker once and did not design a follow-up study. Fourth, the acceptance of AD complications, rehabilitation expectations, and other clinical outcome indicators were important. However, due to the lack of specific evaluation methods, we did not conduct an investigation. Moreover, convenience sampling was used and most of the data were self-reported. It was unable to avoid potential selection bias. Finally, this was a before-and-after comparison study. We did not randomize the patients, which weaken the conclusions that we can draw. A larger controlled trial is warranted to evaluate the effectiveness of such an approach and to measure the change in behavior over a longer term.

## 6. Conclusions

In view of the complexity of decision-making in AD, this study shows that the use of SDM can reduce decision-making conflict, improve decision-making participation, and improve decision-making readiness and decision satisfaction, without affecting the choice of surgical methods and complications. It is suggested that SDM should be rationally incorporated into the process of informed consent of AD. Income, education level, and residence are the influencing factors of decision-making conflict. It is necessary to improve the family’s economic burden by strengthening medical insurance, and ensuring the readability and objectivity of the content of PtDA to improve decision-making conflicts.

## Data Availability

The datasets used and/or analyzed during the current study are available from the corresponding author on reasonable request.

## Supplementary Material

Supplementary material associated with this article can be found, in the online version, at https://doi.org/10.31083/j.rcm2408244.

## References

[1] Mussa FF, Horton JD, Moridzadeh R, Nicholson J, Trimarchi S, Eagle KA (2016). Acute Aortic Dissection and Intramural Hematoma: A Systematic Review. *The Journal of the American Medical Association*.

[2] Zhang D, Zhou Y, Liu J, Hu K, Zhu L, Wu Q (2021). Status Quo of Family Agency Decision-making Before Aortic Dissection Operations and Its Influencing Factors. *Nursing Journal of Chinese People’s Liberation Army*.

[3] Chen Y, Huang S (2015). Experiences of being informed of patients with aortic dissection during the early phase of the disease: a qualitative study. *Chinese Journal of Practical Nursing*.

[4] Zhang D, Zhou Y, Liu J, Hu K, Zhu L, Wu Q (2022). Factors associated with preoperative decision-making in patients with aortic dissection. *Journal of Nursing Science*.

[5] Isselbacher EM, Preventza O, Hamilton Black J, Augoustides JG, Beck AW, Bolen MA (2022). 2022 ACC/AHA Guideline for the Diagnosis and Management of Aortic Disease: A Report of the American Heart Association/American College of Cardiology Joint Committee on Clinical Practice Guidelines. *Circulation*.

[6] Tonelli MR, Sullivan MD (2019). Person-centred shared decision making. *Journal of Evaluation in Clinical Practice*.

[7] Hoefel L, O’Connor AM, Lewis KB, Boland L, Sikora L, Hu J (2020). 20th Anniversary Update of the Ottawa Decision Support Framework Part 1: A Systematic Review of the Decisional Needs of People Making Health or Social Decisions. *Medical Decision Making*.

[8] Wilson CD, Probe RA (2020). Shared Decision-making in Orthopaedic Surgery. *The Journal of the American Academy of Orthopaedic Surgeons*.

[9] Tanner NT, Silvestri GA (2019). Shared Decision-making and Lung Cancer Screening: Let’s Get the Conversation Started. *Chest*.

[10] Probst MA, Noseworthy PA, Brito JP, Hess EP (2018). Shared Decision-Making as the Future of Emergency Cardiology. *The Canadian Journal of Cardiology*.

[11] Lauck SB, Lewis KB, Borregaard B, de Sousa I (2021). “What Is the Right Decision for Me?” Integrating Patient Perspectives Through Shared Decision-Making for Valvular Heart Disease Therapy. *The Canadian Journal of Cardiology*.

[12] Meng X, Han J, Wang L, Wu Q (2021). Aortic dissection during pregnancy and postpartum. *Journal of Cardiac Surgery*.

[13] Elwyn G, O’Connor A, Stacey D, Volk R, Edwards A, Coulter A (2006). Developing a quality criteria framework for patient decision aids: online international Delphi consensus process. *British Medical Journal*.

[14] Coulter A, Stilwell D, Kryworuchko J, Mullen PD, Ng CJ, van der Weijden T (2013). A systematic development process for patient decision aids. *BMC Medical Informatics and Decision Making*.

[15] Sabe AA, Percy ED, Kaneko T, Plichta RP, Hughes GC (2021). When to Consider Deferral of Surgery in Acute Type A Aortic Dissection: A Review. *The Annals of Thoracic Surgery*.

[16] O’Connor AM (1995). Validation of a decisional conflict scale. *Medical Decision Making*.

[17] Wang L, Chen Y, Cui J, Fang H, Liu H, Liao Z (2019). Reliability and validity testing of the Chinese version of Decisional Conflict Scale in patients making decision for rectal cancer surgery. *Journal of Nursing Science*.

[18] Bennett C, Graham ID, Kristjansson E, Kearing SA, Clay KF, O’Connor AM (2010). Validation of a preparation for decision making scale. *Patient Education and Counseling*.

[19] Li Y (2017). Construction and Application of Treatment Decision Aids for Early-Stage Primary Liver Cancer Patients [master’s thesis]. *The Second Military Medical University*.

[20] Xu X (2010). Developing a scale to survey patient’s satisfaction with participation in medical decision-making. *master’s thesis*.

[21] Degner LF, Sloan JA, Venkatesh P (1997). The Control Preferences Scale. *The Canadian Journal of Nursing Research*.

[22] Peng x (2016). Study on the status and influencing factors of attitude to participate in decision-making about treatment and nursing care in breast cancer patients [master’s thesis]. *Huazhong University of Science and Technology*.

[23] Stacey D, Légaré F, Boland L, Lewis KB, Loiselle MC, Hoefel L (2020). 20th Anniversary Ottawa Decision Support Framework: Part 3 Overview of Systematic Reviews and Updated Framework. *Medical decision making*.

[24] Wang S, Lu Q, Ye Z, Liu F, Yang N, Pan Z (2022). Effects of a smartphone application named “Shared Decision Making Assistant” for informed patients with primary liver cancer in decision-making in China: a quasi-experimental study. *BMC Medical Informatics and Decision Making*.

[25] Jönsson M, Berg SK, Missel M, Palm P (2021). Am I going to die now? Experiences of hospitalisation and subsequent life after being diagnosed with aortic dissection. *Scandinavian Journal of Caring Sciences*.

[26] Lipstein EA, Lindly OJ, Anixt JS, Britto MT, Zuckerman KE (2016). Shared Decision Making in the Care of Children with Developmental and Behavioral Disorders. *Maternal and Child Health Journal*.

[27] Elf M, Fröst P, Lindahl G, Wijk H (2015). Shared decision making in designing new healthcare environments-time to begin improving quality. *BMC Health Services Research*.

[28] Evangelista A, Isselbacher EM, Bossone E, Gleason TG, Eusanio MD, Sechtem U (2018). Insights From the International Registry of Acute Aortic Dissection: A 20-Year Experience of Collaborative Clinical Research. *Circulation*.

[29] Eckman MH, Costea A, Attari M, Munjal J, Wise RE, Knochelmann C (2018). Shared decision-making tool for thromboprophylaxis in atrial fibrillation - A feasibility study. *American Heart Journal*.

[30] Orom H, Biddle C, Waters EA, Kiviniemi MT, Sosnowski AN, Hay JL (2020). Causes and consequences of uncertainty about illness risk perceptions. *Journal of Health Psychology*.

[31] Kostick KM, Bruce CR, Minard CG, Volk RJ, Civitello A, Krim SR (2018). A Multisite Randomized Controlled Trial of a Patient-Centered Ventricular Assist Device Decision Aid (VADDA Trial). *Journal of Cardiac Failure*.

[32] Schulz PJ, Pessina A, Hartung U, Petrocchi S (2021). Effects of Objective and Subjective Health Literacy on Patients’ Accurate Judgment of Health Information and Decision-Making Ability: Survey Study. *Journal of Medical Internet Research*.

[33] Xiao L, Peng M, Liu Y, Zhang L (2021). Information, deliberation, and decisional control preferences for participation in medical decision-making and its influencing factors among Chinese cancer patients. *Health Expectations*.

[34] Boss EF, Mehta N, Nagarajan N, Links A, Benke JR, Berger Z (2016). Shared Decision Making and Choice for Elective Surgical Care: A Systematic Review. *Otolaryngology–head and Neck Surgery*.

[35] Stalnikowicz R, Brezis M (2020). Meaningful shared decision-making: complex process demanding cognitive and emotional skills. *Journal of Evaluation in Clinical Practice*.

[36] Alden DL, Friend J, Chun MBJ (2013). Shared decision making and patient decision aids: knowledge, attitudes, and practices among Hawai’i physicians. *Hawai’i Journal of Medicine & Public Health*.

[37] Matlock DD, McIlvennan CK, Thompson JS, Morris MA, Venechuk G, Dunlay SM (2020). Decision Aid Implementation among Left Ventricular Assist Device Programs Participating in the DECIDE-LVAD Stepped-Wedge Trial. *Medical Decision Making*.

[38] Zha Z, Pan Y, Zheng Z, Wei X (2022). Prognosis and Risk Factors of Stroke After Thoracic Endovascular Aortic Repair for Stanford Type B Aortic Dissection. *Frontiers in Cardiovascular Medicine*.

[39] Kunneman M, Branda ME, Hargraves IG, Sivly AL, Lee AT, Gorr H (2020). Assessment of Shared Decision-making for Stroke Prevention in Patients With Atrial Fibrillation: A Randomized Clinical Trial. *JAMA Internal Medicine*.

[40] Zheng H, Hu J, Dong B, Yang Y (2018). Research progress of theoretical models related to sharing decision-making between doctors, nurses and patients. *Chinese Nursing Management*.

[41] Ma Q, Ge H, Deng Y (2015). Influence factors analysis of the hospitalization cost for the acute aortic dissection patients. *Chinese Journal of Critical Care Medicine*.

[42] Li Z, Tian Y, Gong Z, Qian L (2021). Health Literacy and Regional Heterogeneities in China: A Population-Based Study. *Frontiers in Public Health*.

