# Supplementary material for: The Impact of Shared Decision-Making on the Quality of Decision Making in Aortic Dissection: A before-and-after Comparison Study
Source: Rev Cardiovasc Med. 2023 Aug 24;24(8):244. doi: 10.31083/j.rcm2408244 (PMC11266772; doi:10.31083/j.rcm2408244)

Patient decision aid for aortic dissection

Aortic dissection (AD), as an acute and critical disease in cardiovascular surgery, requires surgical treatment. However, surgery is not a routine experience for patients. This PtDA purpose is to improve your disease knowledge level， decision-making readiness, and make high-quality decisions under the guidance of doctors.

This tool will help you if you meet the following conditions:
1 non emergency surgery
2 no surgical decision has been made
3 further understanding of AD

First step: Identify current decision needs

- The current classification of patients with AD is: (Stanford A Stanford B). AD have rapid onset, diverse symptoms and difficult treatment. The delay in preoperative decision-making does not only affect the treatment but also have a postoperative impact.

- Patient is admitted to cardiac surgery (Ward ICU), Whether there are nervous system symptoms such as syncope and irritability at admission (YES NO), Whether sedatives are used (YES NO).

- Who is the surgical decision-maker (patient proxy decision-maker).

- Which decision-making method do you prefer (active decision-making shared decision-making passive decision-making).

Patient decision aid for aortic dissection

Second step： Provide decision-making information support

- Aortic dissection is a life-threatening condition caused by a tear in the intimal layer of the aorta or bleeding within the aortic wall, resulting in the separation (dissection) of the layers of the aortic wall.
- The incidence is about 3/100,000 per year.
- Mortality rates for untreated type A dissection versus type B dissection are as follows:

	24h	48h	2 weeks	30 days
Stanford A	25%	50%(The fatality rate increases by 1% per hour within 48 hours)	90%	
Stanford B	<20%			<85%

- The risk factors are individual differences
  - male
  - age （51.1±10.9）
  - hypertension （50.1%-75.9%）
  - atherosclerosis
  - smoking
  - connective tissue disease (Marfan syndrome is common)
  - congenital cardiovascular disease
  - injure
  - other

Physical examination

- ECG
- blood, urine
- CT
- CTA
- magnetic resonance
- echocardiography
- Angiography, etc

Patient decision aid for aortic dissection

- **In the modern treatment of AD, drug treatment is the basis and runs through the whole treatment process,**

  Surgery and endovascular surgery are fundamental,

  Hybrid surgery is fusion.

- **The in-hospital mortality of patients with AD who underwent early surgical treatment was 13%, and the mortality of drug treatment was 67%.**

- **Principle:**

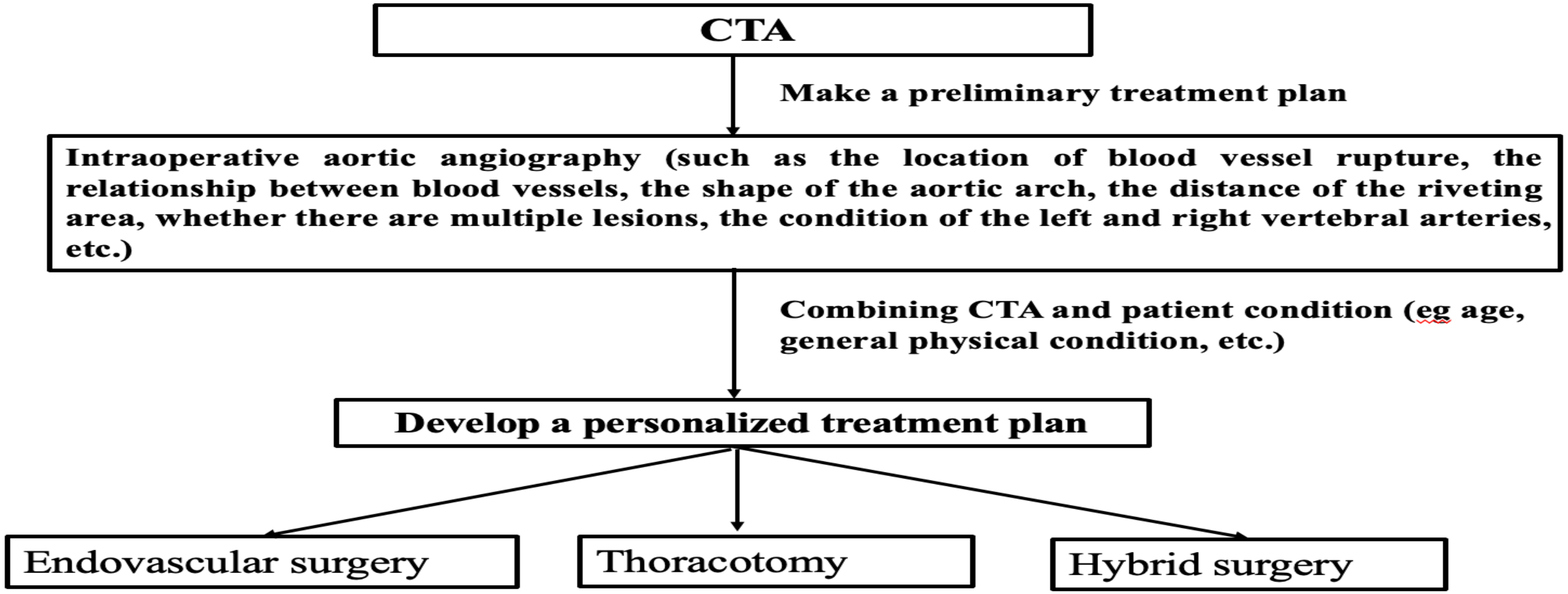

Patient decision aid for aortic dissection

The choice of treatment method shall be determined by the doctor according to the patient's condition.

Treatment	Endoluminal surgery
Principle	Through arterial puncture or small incision, the covered stent is implanted into the aorta to isolate the lumen and restore blood supply.
Indications/ Prerequisites	Healthy aortic wall, long enough anchorage area, etc
Common risks	Retrograde AAD (most severe, 1.4% - 10.0%),<br>Endoleak (9.7%),<br>Neurological complications (such as stroke, 1% - 3%)<br>Paraplegia (less than 1%)<br>The annual reoperation rate was about 30.6%, etc
Benefit	Less trauma<br>Less complications<br>Fast recovery, etc

Patient decision aid for aortic dissection

The choice of treatment method shall be determined by the doctor according to the patient's condition.

Treatment	Thoracotomy
Principle	Prevent aortic rupture and cardiac tamponade, correct aortic regurgitation, and improve hemodynamics.
Indications/ Prerequisites	It is only applicable to ascending aortic dissection and a few descending aortic dissections with serious complications
Common risks	Large trauma, high surgical risk and long postoperative recovery time<br>Acute respiratory insufficiency (most common, 5% - 15%)<br>Neurological complications (4% - 30%)<br>Renal failure (5% - 12%)<br>hemorrhage<br>Infection (12%)<br>Reoperation (the incidence of reoperation intervention 10 years after operation is about 20%) , etc
Benefit	The specific advantages and disadvantages are weighed by the doctor

Patient decision aid for aortic dissection

The choice of treatment method shall be determined by the doctor according to the patient's condition.

Treatment	Hybrid surgery	Conservative treatment
Principle	The combination of thoracotomy and endovascular surgery.	Depressurize, sedate, control heart rate, reduce the shear force of aortic wall and avoid dissection rupture.
Indications/ Prerequisites	It is more suitable for the elderly and high-risk patients with other organ diseases	It is generally not recommended unless the patient is too old to tolerate the operation or the patient and his family are willing to treat conservatively, etc
Common risks	The long-term effect is uncertain, and the patient needs to be exposed to the radiation environment<br>Endoleak (0% - 15%)<br>Sandwich reverse tear, etc	Poor effect<br>Unable to treat the disease, etc
Benefit	Relatively small trauma and rapid recovery<br>Good brain protection<br>Relatively few complications, etc	The cost is relatively low, etc

Patient decision aid for aortic dissection

The choice of treatment method shall be determined by the doctor according to the patient's condition.

Treatment	Thoracotomy	endoluminal surgery	Hybrid surgery	Conservative treatment
Exercise rehabilitation	All patients with aortic dissection should avoid strenuous activities (such as competitive sports, etc.), and aerobic activities are recommended. Resistance breathing activities should be avoided in patients at risk for recurrent pneumothorax.Patients are encouraged to perform light physical activities, such as walking, cycling, planting flowers, etc. It is generally recommended that the weight of lifting heavy objects should not exceed 50% of their body weight. Exercise 3-5 times/day.			
Cost	The cost of surgery for patients with aortic dissection varies from person to person. We only list the cost of surgery and do not involve the cost of later medication. The specific cost is unclear. (Taking stent surgery as an example, thoracic aortic stent-graft endovascular isolation is about 150,000 RMB, abdominal aortic stent-graft endovascular isolation is more than 80,000 RMB, and ascending aorta replacement in open surgery is about 150,000 RMB, Bentall, Sun's surgery, hybrid surgery at least 200,000 RMB).			
Medical insurance policy	The provincial medical insurance reimbursement is about 30%-40%, and the reimbursement ratio of Wuhan medical insurance is higher than that of the city medical insurance.（The expenses of some drugs and surgical consumables need to be paid in full, so the final reimbursement amount and proportion are slightly different）			

Patient decision aid for aortic dissection

Thoracotomy

- **Aortic root treatment**
  - Bentall: Aortic valve replacement——ascending aorta replacement（Valved）——Coronary artery transplantation
  - David: Preservation of aortic valve——Reconstruction of aortic sinus——ascending aorta replacement——Coronary artery transplantation
  - ascending aorta replacement : Separation of ascending aorta——Suture artificial blood vessel
- **Aortic arch treatment**
  - The single bifurcation vessels were anastomosed to the brachiocephalic trunk, left common carotid artery and left subclavian artery respectively
  - Island anastomosis
  - Intraoperative three-branch stent anastomosis
- **Descending aorta treatment**

Patient decision aid for aortic dissection

Endoluminal surgery

- **Hybrid surgery**
- **Fenestration technology**
  - **Extracorporeal fenestration:** Relatively good cerebral perfusion; less head and neck operations, low incidence of cerebral infarction; but high technical requirements and high requirements for the positioning of branch arteries.
  - In Situ Fenestration: Most ISF cases were performed at the arch level, both in the elective and emergency setting. Various mechanical and physical means were used to perform ISF. High technical success rates with satisfactory short-term results were reported but long-term outcomes are lacking.
- **Endografts with parallel stents, namely CHIMPS (Chimney, Periscope, Sandwich):** Minimally invasive surgery, reduce trauma and shorten operation time, but there are risks such endoleak and cerebral infarction.

Patient decision aid for aortic dissection

- Are you nervous or anxious? YES NO
- Have you found support from your family? YES NO
- What your family thinks about treatment options? Positive Unclear Negative

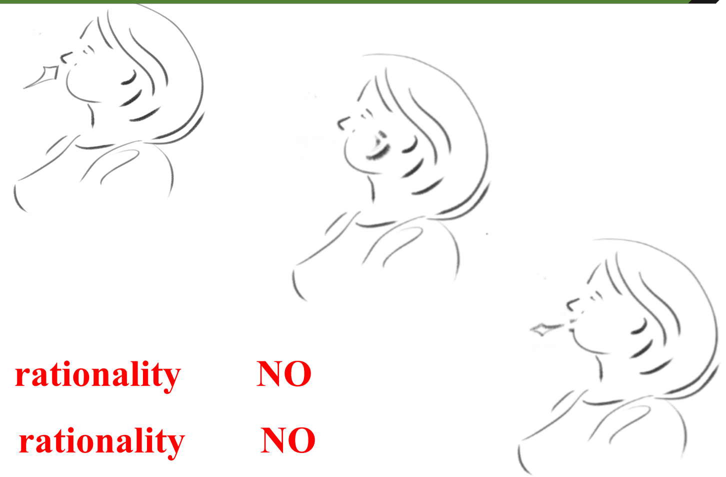

Third step : Clarify the values of decision-makers

- I hope the patient can be out of danger after surgery. YES rationality NO
- I hope that the patient‘s discomfort will be relieved after the surgery. YES rationality NO
- I hope surgical treatment can ensure family integrity and subsequent family quality of life. YES rationality NO
- My acceptance of the risks that arise during surgery. can‘t accept rationality acceptable
- My acceptance of the gap between the expected outcome of the surgery and the actual recovery of the patient. can‘t accept rationality acceptable
- My acceptance of complications. can‘t accept rationality acceptable

Fourth step: Guide decision making

- Do you know anything about aortic dissection? YES NO
- Have you carefully considered the treatment plan for your personal situation? YES NO
- Are you satisfied with your current treatment plan? YES NO
- What is your role in decision making ? active decision-making shared decision-making passive decision-making
- What is your final choice ? Thoracotomy endoluminal surgery Hybrid surgery Conservative treatment Give up

Do you have any other questions, if any:

Patient decision aid for aortic dissection

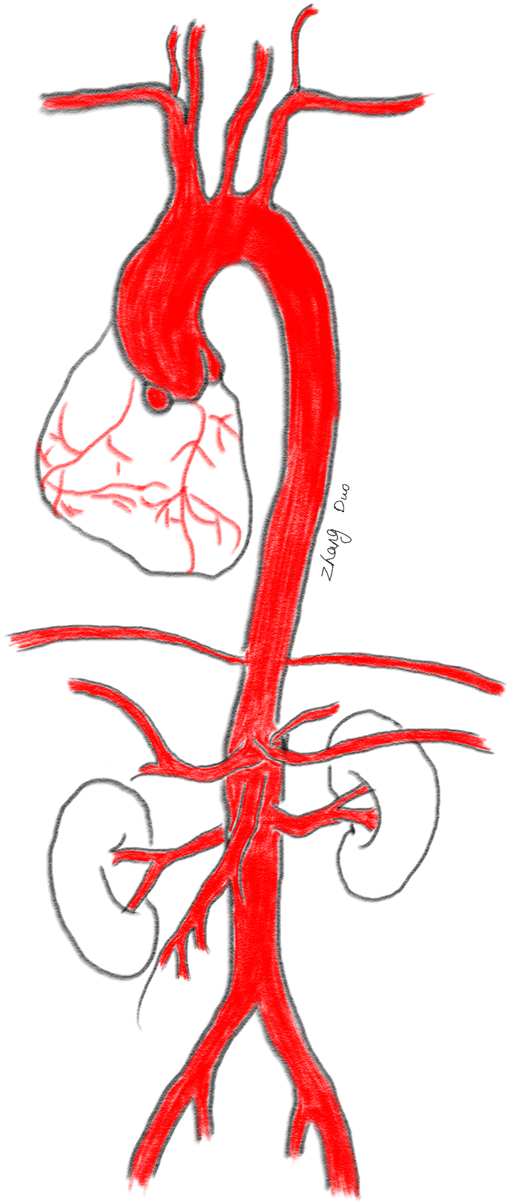

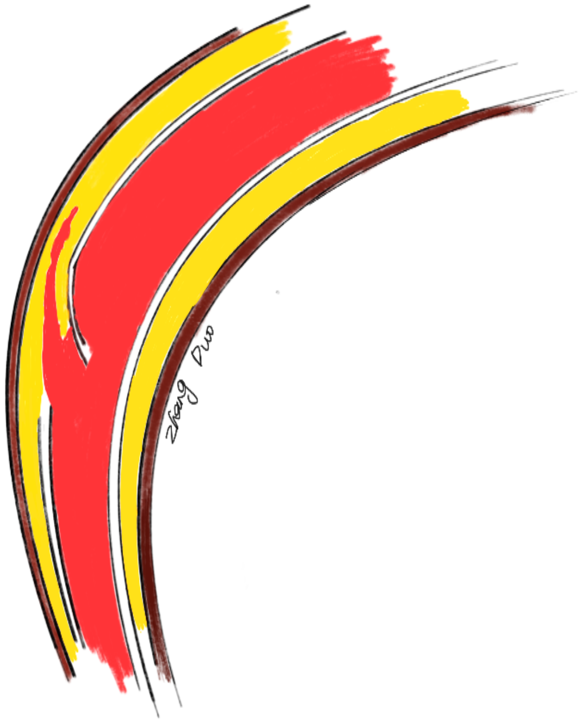

Patient decision aid for aortic dissection

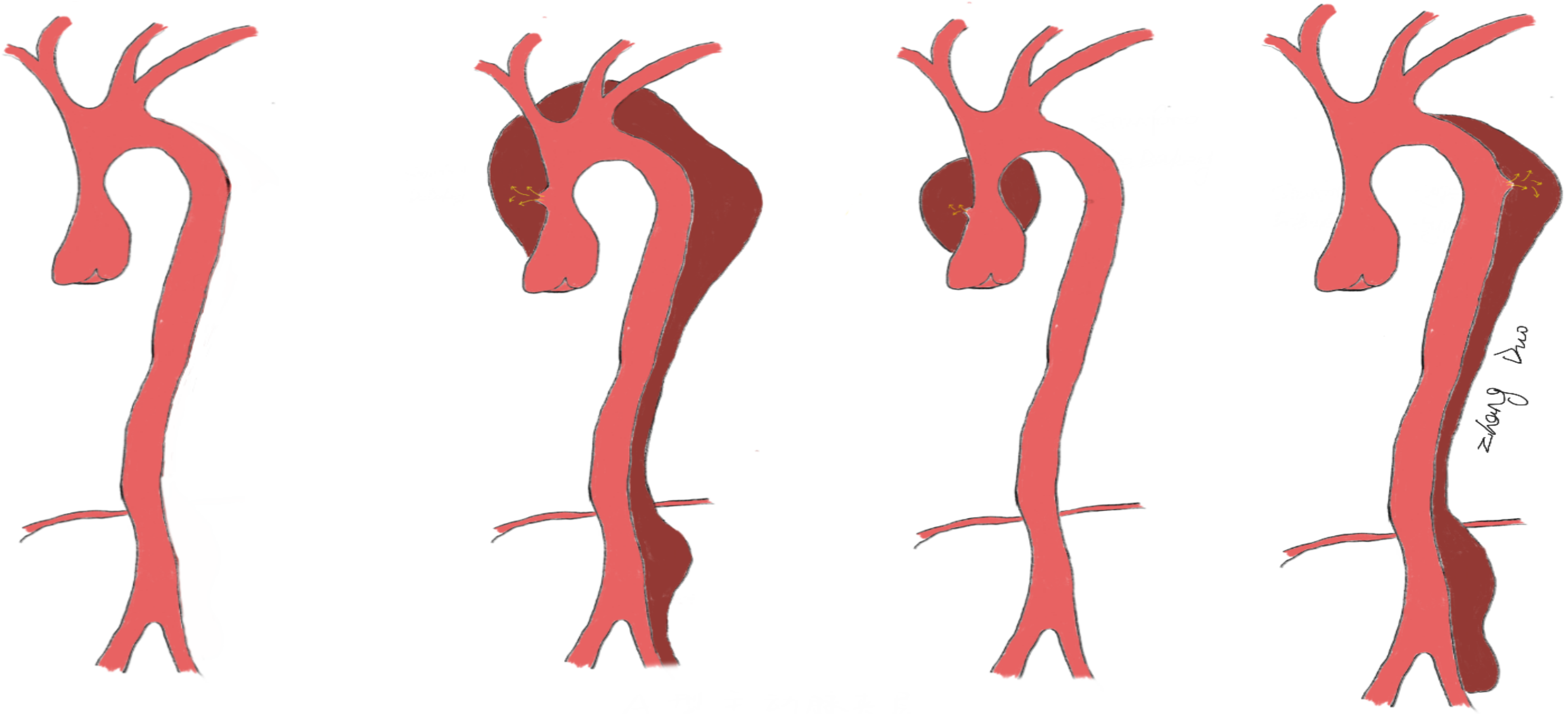

DEBAKEY	I	II	III
STANFORD	A	A	B

Patient decision aid for aortic dissection

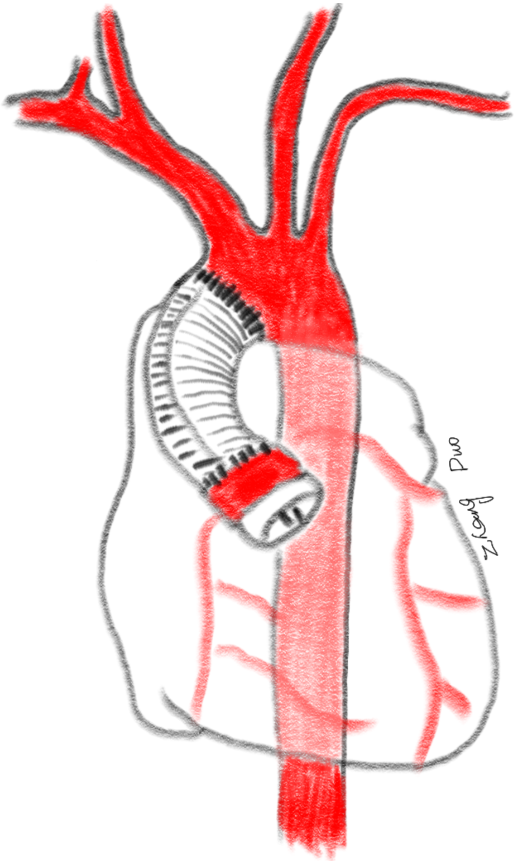

Wheat

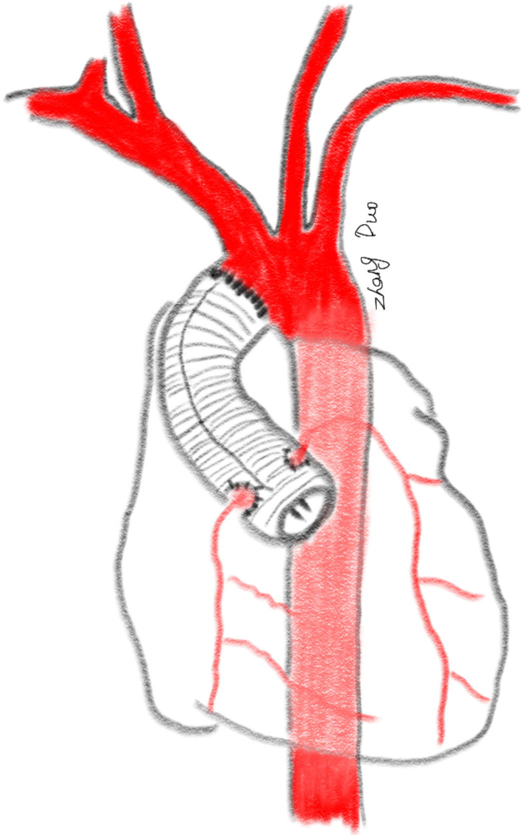

Bentall

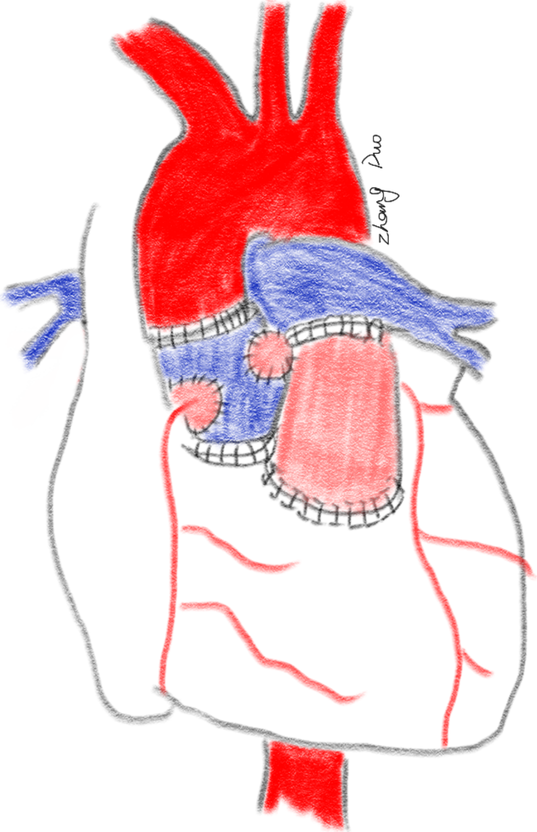

Ross

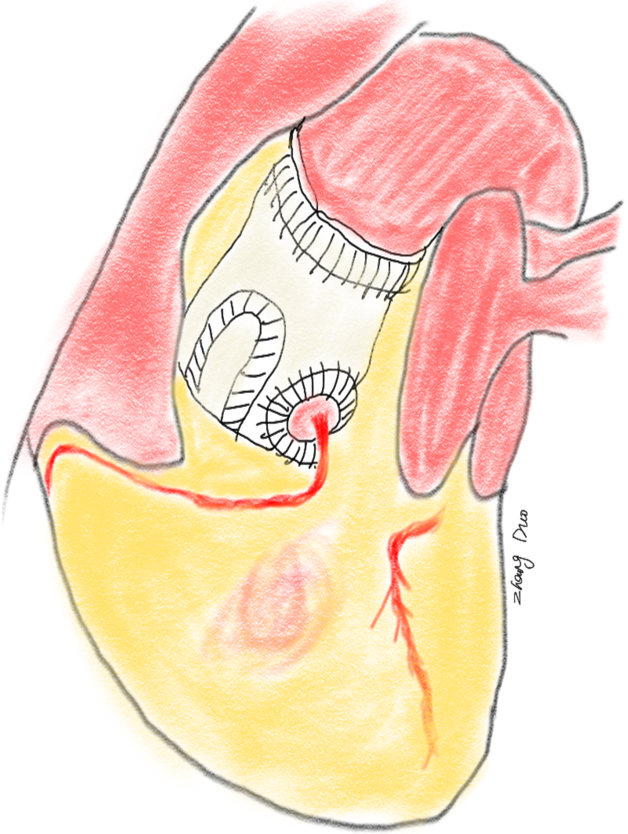

David

Patient decision aid for aortic dissection

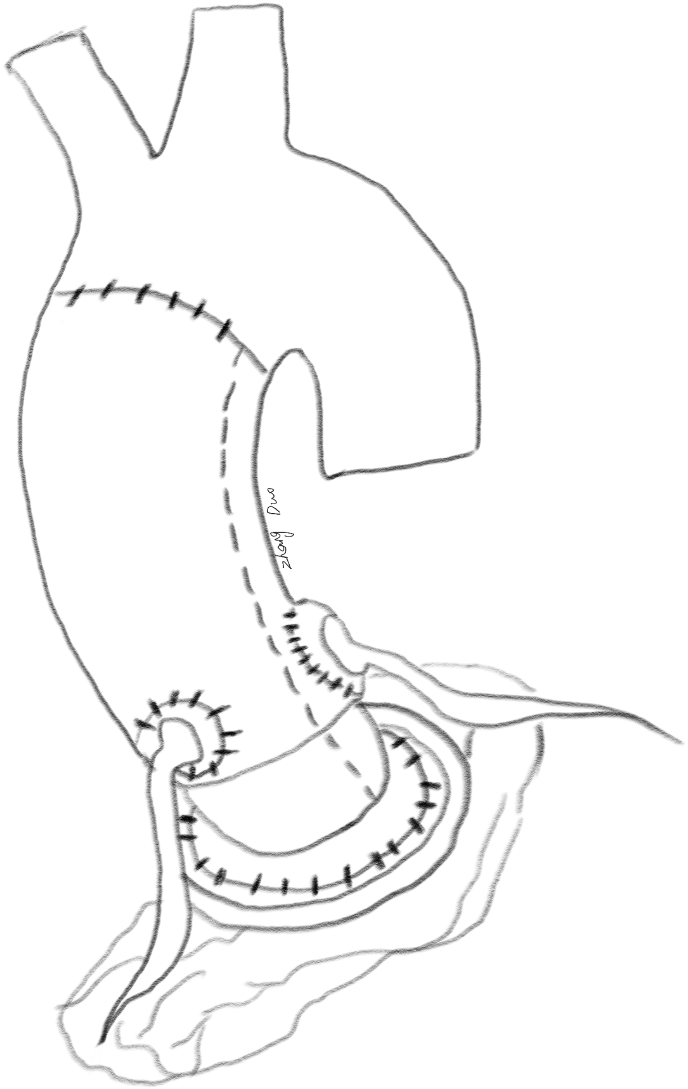

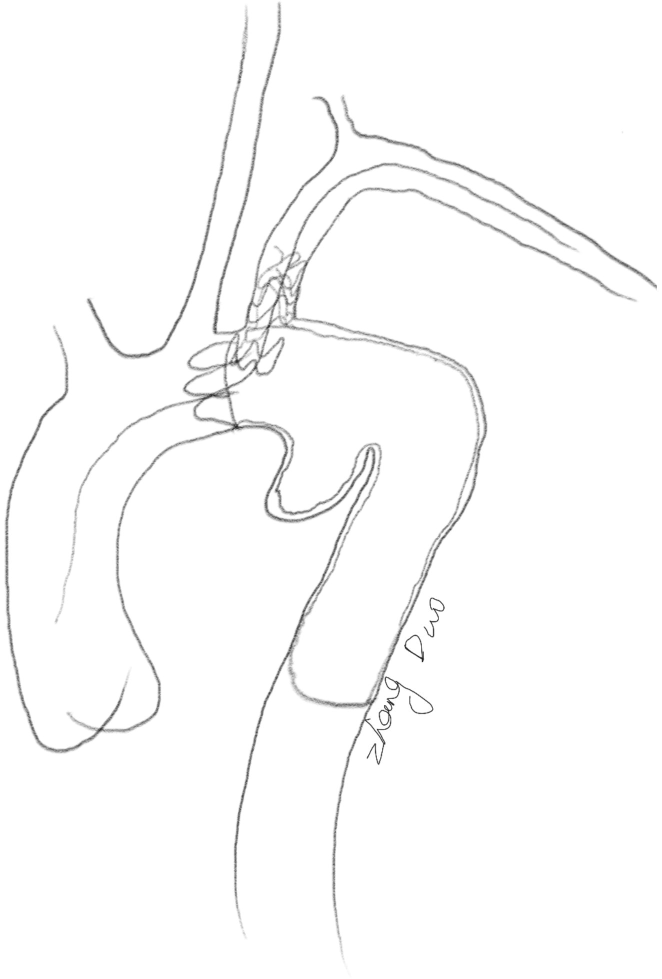

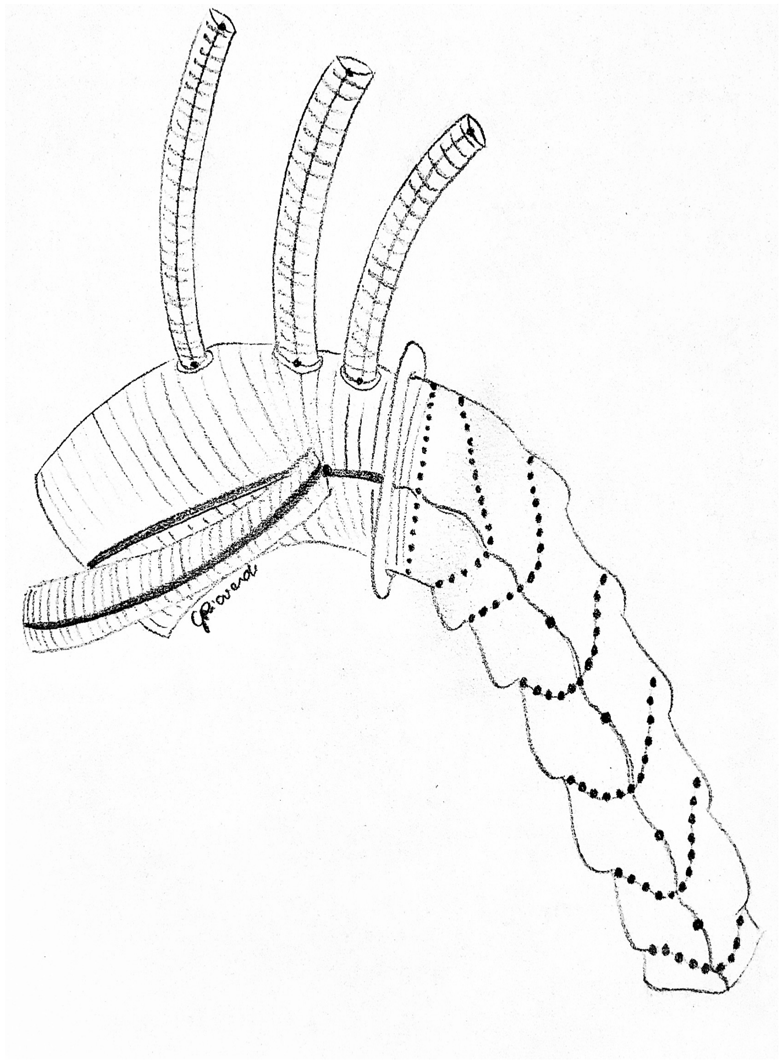

Patient decision aid for aortic dissection

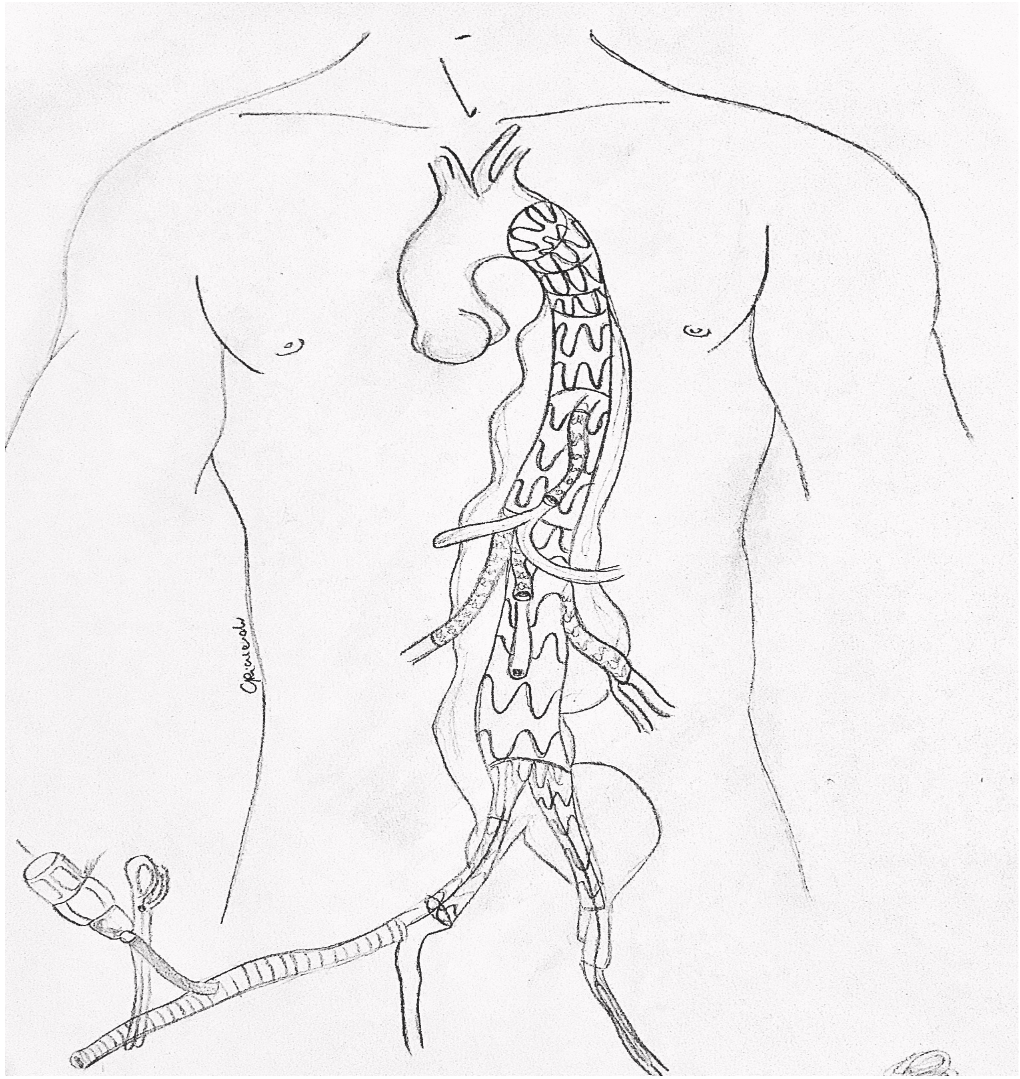

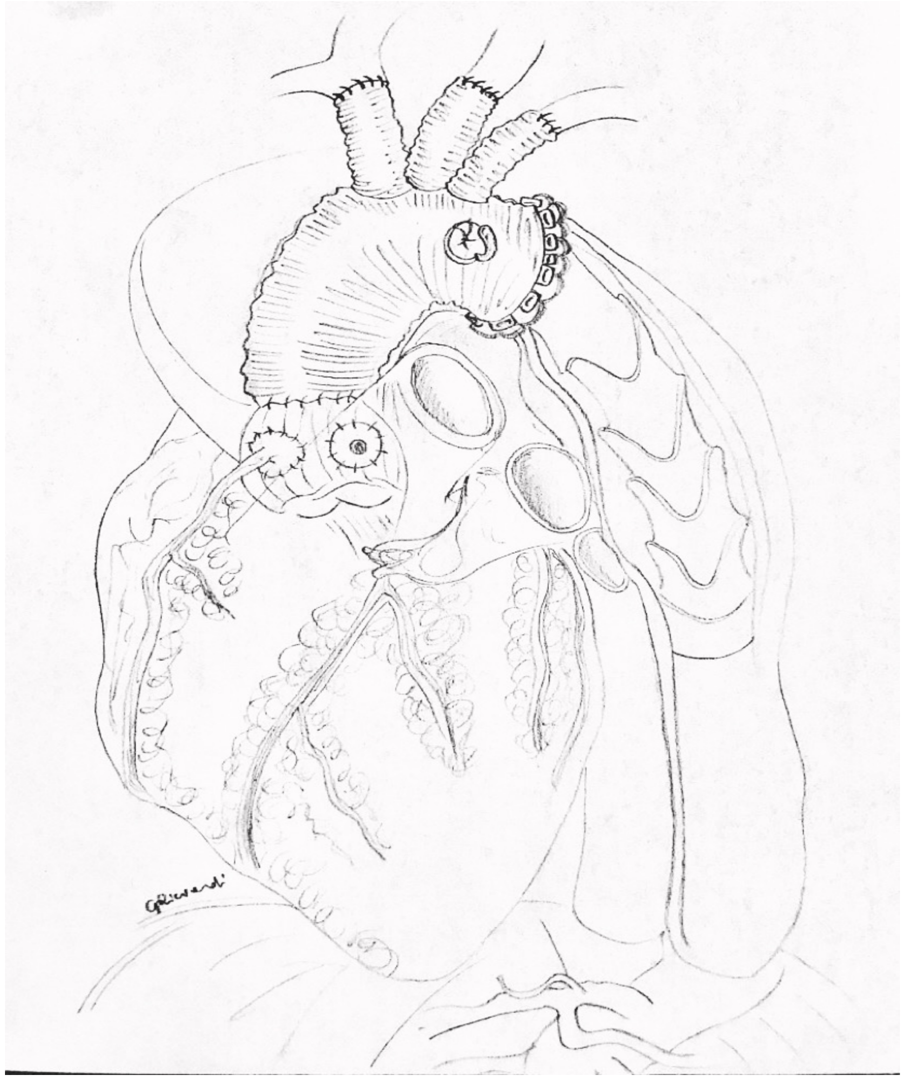

The above hand drawing has been authorized by Gabriella Ricciardi, Department of Cardiac Surgery, Leiden Universitair Medisch Centrum, Leiden, Netherlands

Patient decision aid for aortic dissection

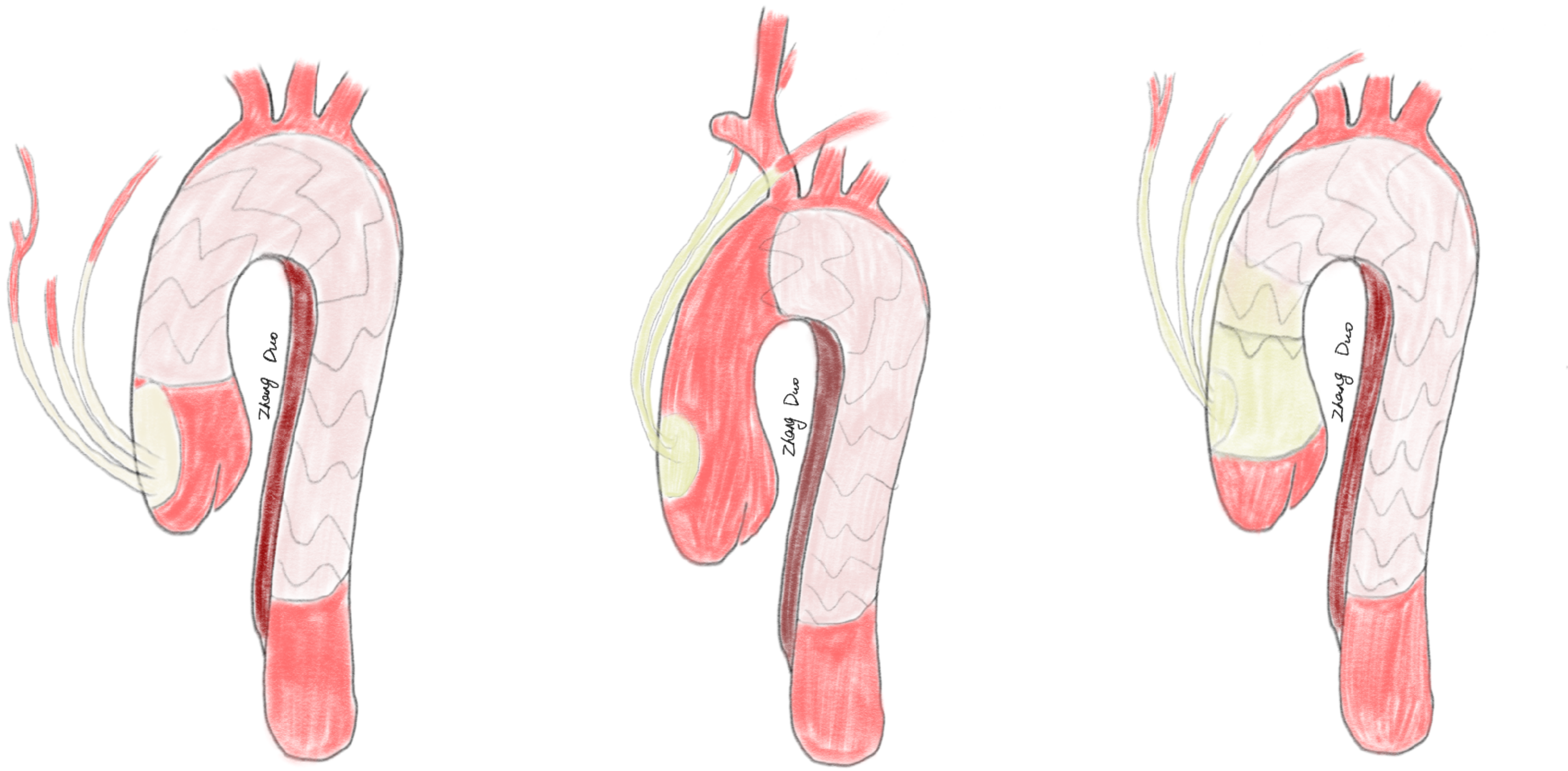

Patient decision aid for aortic dissection

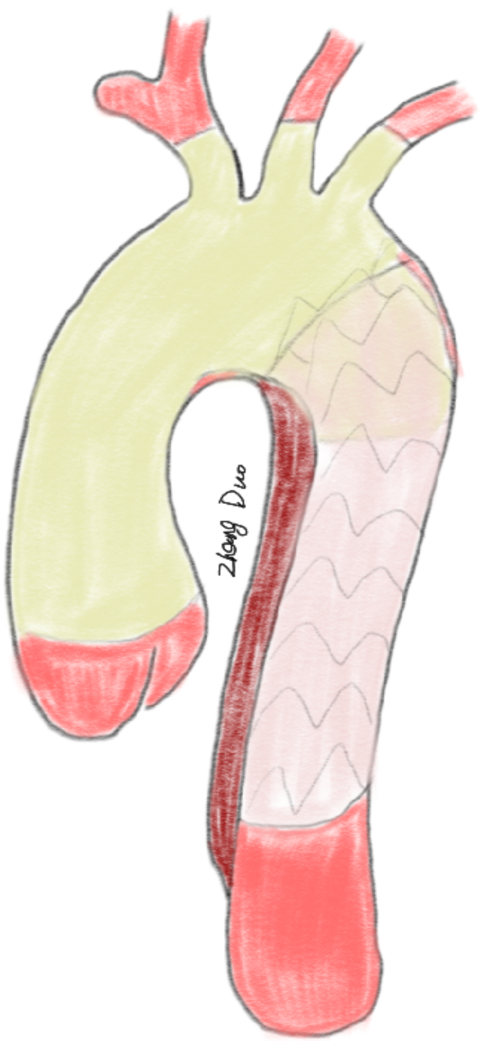

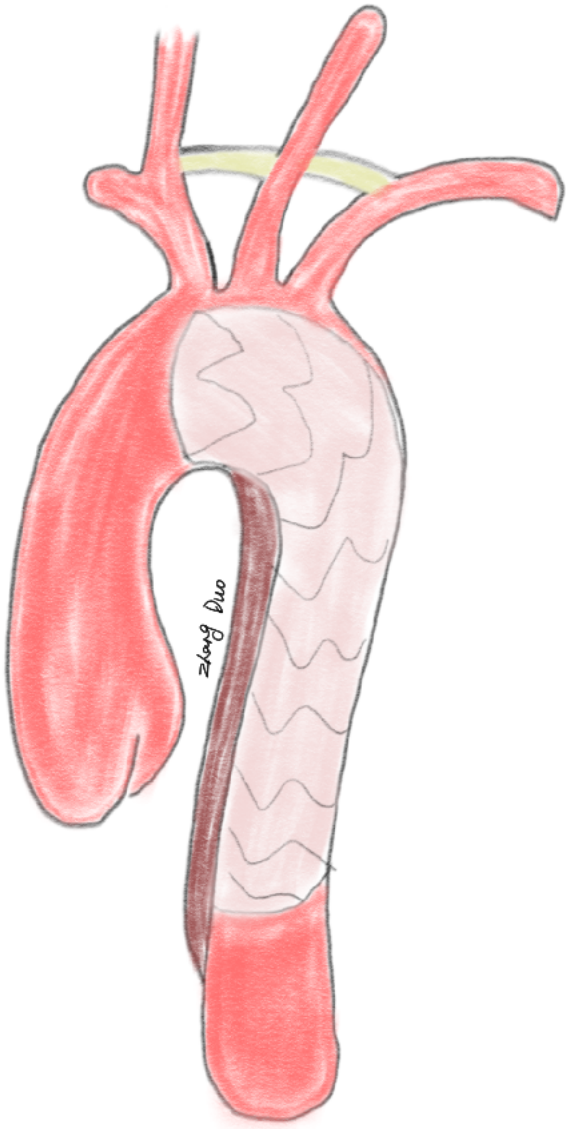

Supplement: Supplementary file 1 [file 2153-8174-24-8-244-s1.pdf]
